# Laser-activated nanoparticles for ultrasound/photoacoustic imaging-guided prostate cancer treatment

**DOI:** 10.3389/fbioe.2023.1141984

**Published:** 2023-03-21

**Authors:** Linkang Xiao, Yunfang Wu, Junyong Dai, Weili Zhang, Yang Cao

**Affiliations:** ^1^ Chongqing Key Laboratory of Ultrasound Molecular Imaging, Institute of Ultrasound Imaging, Department of Urology Surgery, Second Affiliated Hospital, Chongqing Medical University, Chongqing, China; ^2^ Chongqing General Hospital, Chongqing, China; ^3^ Chongqing Wanzhou District Maternal and Child Health Hospital, Chongqing, China; ^4^ Chongqing University Cancer Hospital, Chongqing, China

**Keywords:** laser-activated nanoparticles, photothermal therapy (PPT), photoacoustic imaging (PAI), ultrasound, prostate cancer

## Abstract

Prostate cancer (PCa) is the most common malignant tumor in men. Prostate-specific membrane antigen (PSMA), which is overexpressed on the surface of Prostate cancer cells, may serve as a potential therapeutic target. Recently, image-guided and targeted therapy for prostate cancers has attracted much attention by using Prostate-specific membrane antigen targeting nanoparticle. In this study, we produced PSMA-targeted light-responsive nanosystems. These nanosystems of liquid perfluorocarbon cores and polymer shells were loaded with the photosensitizer IR780 and therapeutic drugs paclitaxel. The liquid perfluorocarbon (PFP) in nanoparticles can perform ultrasound-enhanced imaging by liquid-gas transition and promote the deliver and release of paclitaxel. IR780 can perform photothermal therapy (PTT) guided by photoacoustic (PA) imaging. Combination treatment with photothermal therapy and chemotherapy exhibited excellent inhibition of cell proliferation *in vitro* and a significant therapeutic effect *in vivo*. In conclusion, we successfully formulated PSMA-targeted nanosystems with precision targeting and ultrasound/PA dual-modality imaging for anti-tumor effects.

## 1 Introduction

Globally, prostate cancer (PCa) is the second most common cancer type in men and the fifth leading cause of cancer-related death worldwide (Sung et al., 2021; Liu et al., 2022). Although most PCa is indolent or extremely slowly progressing, approximately 20% of patients are diagnosed with the fatal high-risk form, life-threatening (Chang et al., 2014; Adamaki and Zoumpourlis, 2021). The most attractive of the prostate antigens is prostate-specific membrane antigen (PSMA), which is a membrane-bound zinc metalloproteinase mainly expressed by prostate epithelial cells (Ceci et al., 2017). PSMA has been used as a target for targeted therapy or targeted delivery of nanocarriers (Sun et al., 2021; Chen et al., 2022; Guo et al., 2022). Recently, triggerable drug-loaded nanocarriers have been combined with various internal or external stimuli to reduce systemic toxicity (Wang et al., 2018). Internal stimuli are mainly aimed at the tumor microenvironment, such as pH (Zhu and Chen, 2015), hydrogen peroxide (Yang et al., 2017), glutathione (Yuan et al., 2019), etc. External stimuli are mainly physical factors, such as ultrasound (Rapoport, 2016), near-infrared (NIR) light (Tiwari et al., 2018), etc. When perfluoropentane (PFP) reaches a phase-transition temperature of 29°C, it can change from liquid phase to gas phase. Therefore, it can control this phase-transition process by NIR laser irradiation, promoting controlled drug release, and enhanced chemotherapeutic effects (Sheng et al., 2021; Wang et al., 2021; Yang et al., 2022). In addition, the gas bubble changed from liquid agents could exhibit the contrast-enhanced ultrasound imaging for cancer diagnosis (Xie et al., 2018; Waqar et al., 2022).

Although chemotherapy is a common anti-tumor treatment in clinic, some other methods have been developed in prostate cancer treatments. For example, photothermal therapy (PTT) mediated by NIR irradiation of photosensitizers to convert light energy into heat energy can directly destroy tumor tissue (Li et al., 2021; Lv et al., 2021). It showed a great potential for medical application. In PTT, photosensitizer is an important factor affecting photothermal effect. As a derivative of indocyanine green, IR780 has been confirmed to have good photothermal properties. Meanwhile, it can also be used for photoacoustic (PA) imaging (Song et al., 2019; Li et al., 2021; Ren et al., 2021; Wang et al., 2022). PA imaging is a new type of non-invasive, non-radioactive imaging method emerging in recent years (Zackrisson et al., 2014; Steinberg et al., 2019). When the laser emitted from the probe is used to irradiate the biological tissue, the biological tissue containing light-absorbing agents could absorb the laser energy and convert it into heat, which will generate ultrasonic signals for further detection (Moore and Jokerst, 2019; Manohar and Gambhir, 2020; Mallidi et al., 2021).

Here, in this study, FDA-approved poly (lactic-glycolic acid) acid (PLGA), which degraded into carbon dioxide and water, was used as the shell polymer of nanocarriers. After surface modification, the nanocarriers can target the PSMA of PCa. The loading IR780 enable the nanosystems have PA imaging and photothermal capabilities. And the liquid-to-gas transition of PFP within cores after NIR irradiation resulted in the contrast-enhanced ultrasound imaging and controlled release the chemotherapeutic drug paclitaxel. This approach was believed to allow these nanoparticles to have ultrasound/PA dual-mode imaging potential. Moreover, under the guidance of ultrasound/PA imaging, prostate tumors were accurately targeted and killed by combining photothermal and chemotherapy efficiently.

## 2 Material and methods

### 2.1 Materials and reagents

PLGA–COOH (50: 50, MW = 12.3 kDa) was purchased from Jinan Daigang Biomaterial Co. (Shandong, China). Paclitaxel was purchased from Meilun Bio (Dalian, China). Perfluoropentane (PFP), polyvinyl alcohol (PVA), IR780, *N*-(3-dimethyl-aminopropyl)-*N*′-ethylcarbodiimide hydrochloride (EDC), 2-(*N*-morpholino) ethanesulfonic acid (MES monohydrate) and 1,1′-dioctadecyl-3,3,3′,3′ tetramethylindocarbocyanine perchlorate (DiI), and 4′,6-diamidino-2-phenylindole (DAPI) were obtained from Sigma-Aldrich (St. Louis, MO, USA). PSMA monoclonal antibody was purchased from Cell Signaling Technology (USA). Goat anti-human IgG Fc (FITC) was purchased from Abcam (Shanghai, China). Cell Counting Kit-8 (CCK-8) was obtained from Dojindo Molecular Technology (Shanghai, China). RPMI-1640 mediumand fetal bovine serum (FBS) were purchased from Gibco (USA). All other reagents used in this work were of analytical grade and were used as received.

### 2.2 Synthesis of IR780-loaded PFP polymer nanoparticles

50 mg PLGA-COOH, 2 mg IR780, and 1 mg paclitaxel were dissolved in 2 ml dichloromethane. 200 μl PFP was added to the above-mixed solution. The probe sonicator (Sonics and Materials Inc., USA) was used to conduct the first emulsification (60 W, 3 min, 5 s on and 5 s off). Next, 5 ml of 4% PVA aqueous solution was added to the above solution for the second emulsification (45 W, 3 min, 5 s on and 5 s off). 10 ml of isopropanol solution (2%) was added and magnetically stirred for 6 h to remove dichloromethane. Finally, the nanoparticles (PIP NPs) were purified and washed by centrifugation.

### 2.3 PSMA conjugation

Conjugation of PSMA to nanoparticles was performed using carbodiimide chemistry. The prepared PIP NPs was first suspended in 5 ml MES buffer solution (0.1 M, pH 5.5) with a mixture of 3 mg EDC and 10 mg NHS, followed by vigorous incubation on a gentle shaker for 1 h to activate the carboxyl groups. Unreacted EDC and NHS were removed by centrifuge wash. Then, resuspending the pellet in 5 ml of MES buffer solution (0.1 M, pH 8.0). Add an excessive amount of PSMA to the above solution and stir it on a mild shaking table for another 2 h. After the reaction, the unreacted PSMA was removed by centrifugation to obtain PSMA targeted nanoparticles (P-PIP NPs).

### 2.4 Characterization of nanoparticles

The internal structure of NPs was observed by Transmission electron microscopic (TEM, H-7500; Hitachi, Tokyo, Japan). The size and zeta potential were determined using dynamic light scattering (DLS, Malvern Instruments Ltd., UK). The UV–vis absorption spectra were obtained using a UV–vis spectrophotometer (US-2550, Shimadzu, Japan). The phase-transitions of NPs were monitored and recorded under an optical microscope (CKX41; Olympus, Tokyo, Japan). To detect the binding between PSMA and PIP NPs, DiI-labeled NPs were incubated with FITC-labeled goat anti-human IgG and observed by the confocal laser scanning microscopy (CLSM) (Nikon A1, Tokyo, Japan). Flow cytometry analysis (FCM; FACSVantage, BD, USA) was applied to analyze the binding efficiency further. The encapsulation efficiency of IR780 was calculated using UV–vis spectrophotometer. The encapsulation efficiency of paclitaxel was measured by high-performance liquid chromatography (HPLC; ShmadzulC-2-1-AHT; Japan).

### 2.5 *In vitro*photothermal effect and liquid-gas phase-change property of nanoparticles

P-PIP NPs at different concentrations (6.25, 12.5, 25, 50, 75, and 100 μg mL^−1^) in a 96-well plate was irradiated by an 808 nm laser (1 W cm^−2^). The temperature was recorded in real-time using an infrared camera (Fotric 226, Shanghai, China). Next, P-PIP NPs at a fixed concentration (2.5 mg mL^−1^) were irradiated at various irradiation intensities (0.25, 0.5, 0.75, 1.0, and 1.25 W cm^−2^). To examine the liquid-gas phase-change property of P-PIP NPs, the liquid-gas phase-transition of PFP in P-PIP NPs was observed using an optical microscope (IX53; Olympus, Tokyo, Japan).

### 2.6 Targeting efficiency *in vitro*

The PSMA-positive prostate cancer cell line C4-2 was obtained from Chongqing Key Laboratory of Ultrasound Molecular Imaging. C4-2 cells were maintained in RPMI 1640 supplemented with 10% fetal bovine serum at 37°C and 5% CO_2_. C4-2 cells were seeded into glass-bottom cell culture dishes and incubated for 24 h. DiI-labeled nanoparticles with and without PSMA modification were incubated with cells for 4 h, respectively. The dishes were rinsed with phosphate-buffered saline (PBS) three times. The cells in the different groups were washed with PBS three times, fixed with 4% paraformaldehyde for 10 min, and then incubated with DAPI (10 μg mL^−1^, 100 μl) for 8 min. Finally, the dishes were imaged by CLSM. FCM was applied to evaluate the mean fluorescence intensity originating from each group.

### 2.7 Cell viability and apoptosis assay

The cell viability was evaluated by the CCK-8 assay. C4-2 cells (1 × 10^4^ cells per well) were seeded into 96-well plates. After incubation, the cells were divided into four groups: Control, NIR, P-PIP NPs, and P-PIP NPs + NIR. NPs at a fixed concentration (2.5 mg mL^−1^) were co-incubated with cells for 4 h. According to the manufacturer’s instructions, a CCK-8 assay was conducted, and the relative cell viability rate was calculated.

C4-2 cells (5 × 10^5^cells per well) were seeded into a six-well plate and incubator overnight. The cells were divided into four groups: Control, NIR, P-PIP NPs, and P-PIP NPs + NIR. The cells were collected and resuspended in 200 μl of PBS, and FCM was applied to detect and analyze cellular apoptosis.

### 2.8 *In vitro* and in vivoUltrasound and PA imaging

A MyLab90 US diagnostic instrument (Esaote, Genoa, Italy) was used for US imaging. To assess thein vitro liquid–gas phase-transition of PFP, US images of the P-PIP NPs (5.0 mgmL−1) were captured at 0, 1, 2, 3 and 4 min after NIR irradiation. For *in vivo* US imaging, tumor-bearing mice were administered with PIP NPs and P-PIP NPs saline solution intravenously. Then, the US images were collected at different time points.

Forin vitro PA imaging of P-PIP NPs, the samples were first scanned with an excitation wavelength range of 680–970 nm on a PA imaging system (Vevo LAZR, Canada). PA imaging was performed at different concentrations of P-PIP NPs, and the corresponding PA signal was obtained. For *in vivo* PA imaging, tumor-bearing mice were administered with PIP NPs and P-PIP NPs saline solution intravenously. Then, the PA images were collected at different time points (0 h, 6 h, 12 h, and 24 h).

### 2.9 Anti-tumor treatment *in vivo*

A total of 30, male, 4-week-old, weighing approximately 20 g, nude-mice were purchased from Beijing Huafukang Bioscience Co. Inc., China. The animal protocols and experimental procedures regarding animal maintenance and experiments are in strict accordance with the policy of the Institutional Animal Care and Use Committee (IACUC) of Chongqing Medical University. The IACUC has approved this study (NO.2019126). All surgery was performed under pentobarbital sodium injection anesthesia, and all efforts were made to minimize suffering. The xenograft tumor model was established by subcutaneously injecting 0.1 ml of PBS (pH 7.4) containing 2 × 106 tumor cells into the intercostals of lateral abdomen of each mouse. The mice were given free access to water and food and housed in an environment with a 12-h light/dark cycle at 24°C and a relative humidity of 50–70%. The antitumor effect was evaluated on mice bearing prostate cancer xenografts when the subcutaneous tumor reached 100 mm3 in volume.

In total, the tumor-bearing mice were divided randomly into six groups (n = 5 per group): the control group (control), NIR, PIP NPs, P-PIP NPs, PIP NPs + NIR, and P-PIP NPs + NIR. The mice were intravenously injected with 0.2 ml of saline, NPs (5 mgmL−1) or targeted NPs (5 mgmL−1). After 24 h, NIR laser (808 W·cm-2) was used to irradiate the tumor sites. The tumor volume and body weight of each mouse was recorded every 2 days. When the weight of mice dropped below 20% of their initial weight, they were euthanized. All the mice were scarified at day 14 after therapy by cervical dislocation. The major organs as well as tumor tissues were collected. Proliferating cell nuclear antigen (PCNA) and terminal deoxynucleotidyl transferase (TdT) dUTP nick-end labeling (TUNEL) stainings were performed to evaluate tumor cell proliferation and apoptosis. To evaluate the biological toxicity of P-PIP NPs, blood samples were collected for hematological examination, biochemical examination of the liver function assay.

### 2.10 Statistical assay

Each experiment was repeated at least three times. The data are presented as the mean ± SD and statistical analyses were conducted using OriginPro 8.5 software (OriginLab, MA, USA). Differences between two groups were analyzed using unpaired Student’s t-test, whereas differences among three or more groups were assessed by one-way ANOVA and Tukey’s *post hoc* test. A *p*-value less than 0.05 was considered to be statistically significant.

## 3 Results

### 3.1 Synthesis and characterization of P-PIP NPs

**FIGURE 1:**
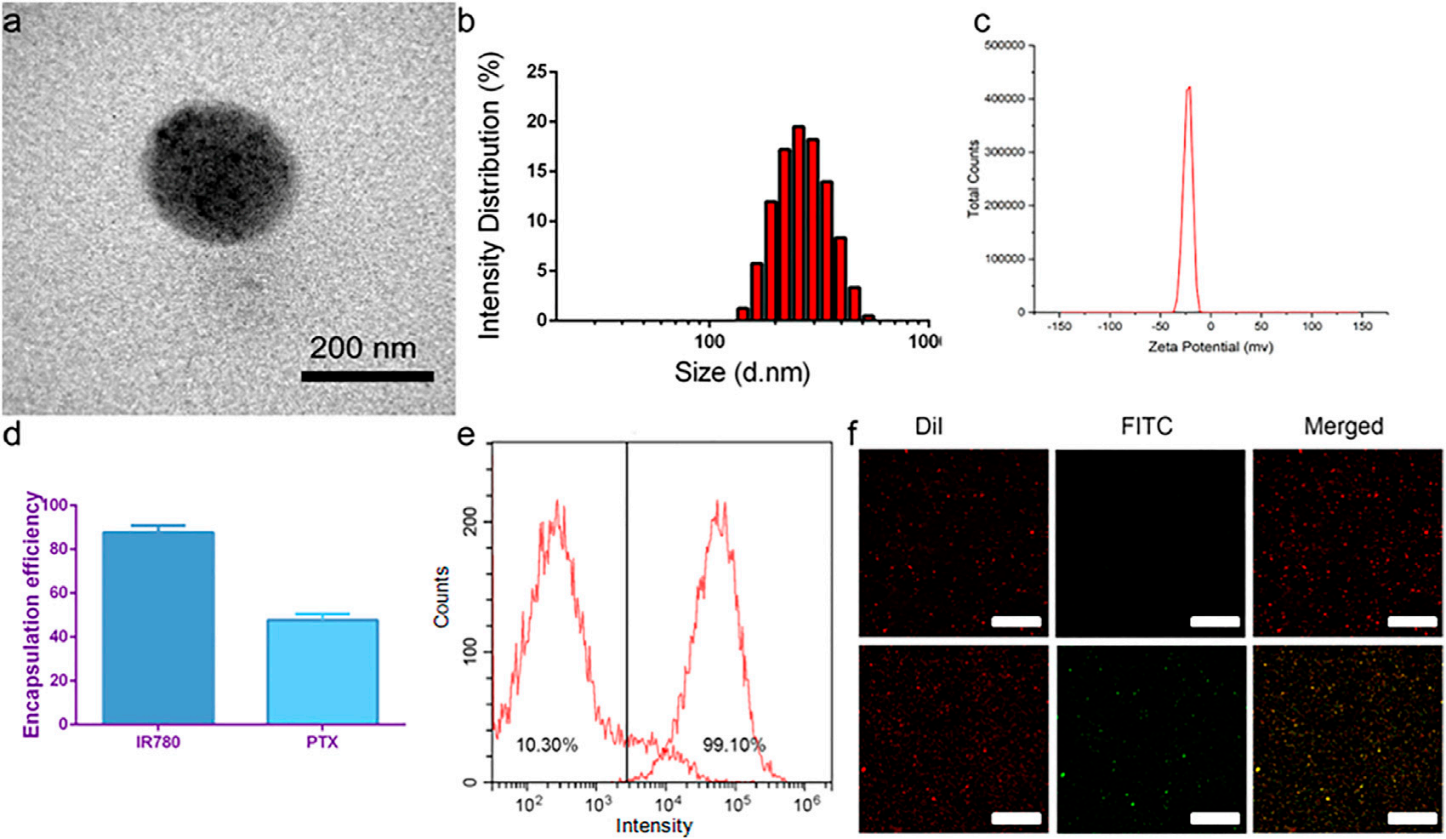
Characterization of P-PIP NPs. **(A)** TEM images, **(B)** size distributions, **(C)** zeta potential, **(D)** encapsulation rates of IR780 and paclitaxel in P-PIP NPs, **(E)** flow-cytometry results of the average binding efficiency **(F)** CLSM images showing preferential binding between FITC-labeled secondary antibody and DiI-labeled P-PIP NPs (Up: PIP NPs; Down: P-PIP NPs). Scale bar: 50 μm.

TEM showed that the targeted nanoparticle encapsulating IR780, paclitaxel and PFP was a uniform spherical structure with a size about 200 nm (Figure 1A). The average size of P-PIP NPs measured by a DLS system was 250 ± 37.5 nm, consistent with the TEM results (Figure 1B). The zeta potential result exhibited an average potential of -12.43 mV (Figure 1C). The encapsulation rates of IR780 and paclitaxel in P-PIP NPs were calculated to be 86.46% and 51.37%, respectively (Figure 1D). A FITC-labeled secondary antibody was used as an indirect fluorescent marker to indicate the binding between PMSA and PLGA nanoparticles. A significant yellow fluorescent signal was observed for PMSA-linked P-PIP NPs compared to the control with pure red light (Figure 1F). FCM showed that the mean fluorescence intensity of PIP NPs and P-PIP NPs were about 10.30% and 99.10%, respectively (Figure 1E), confirming the high degree of conjugation between the secondary antibody and P-PIP NPs. This result indicated that PSMA was successfully attached to the polymer nanoparticles. The efficiency of covalent conjugation was also confirmed with BCA protein assay. The results showed that 36.3 μg of antibodies were successfully bound to 10 mg of PLGA nanoparticle.

### 3.2 *In vitro*photothermal effect and liquid–gas phase-change property of P-PIP NPs

**FIGURE 2:**
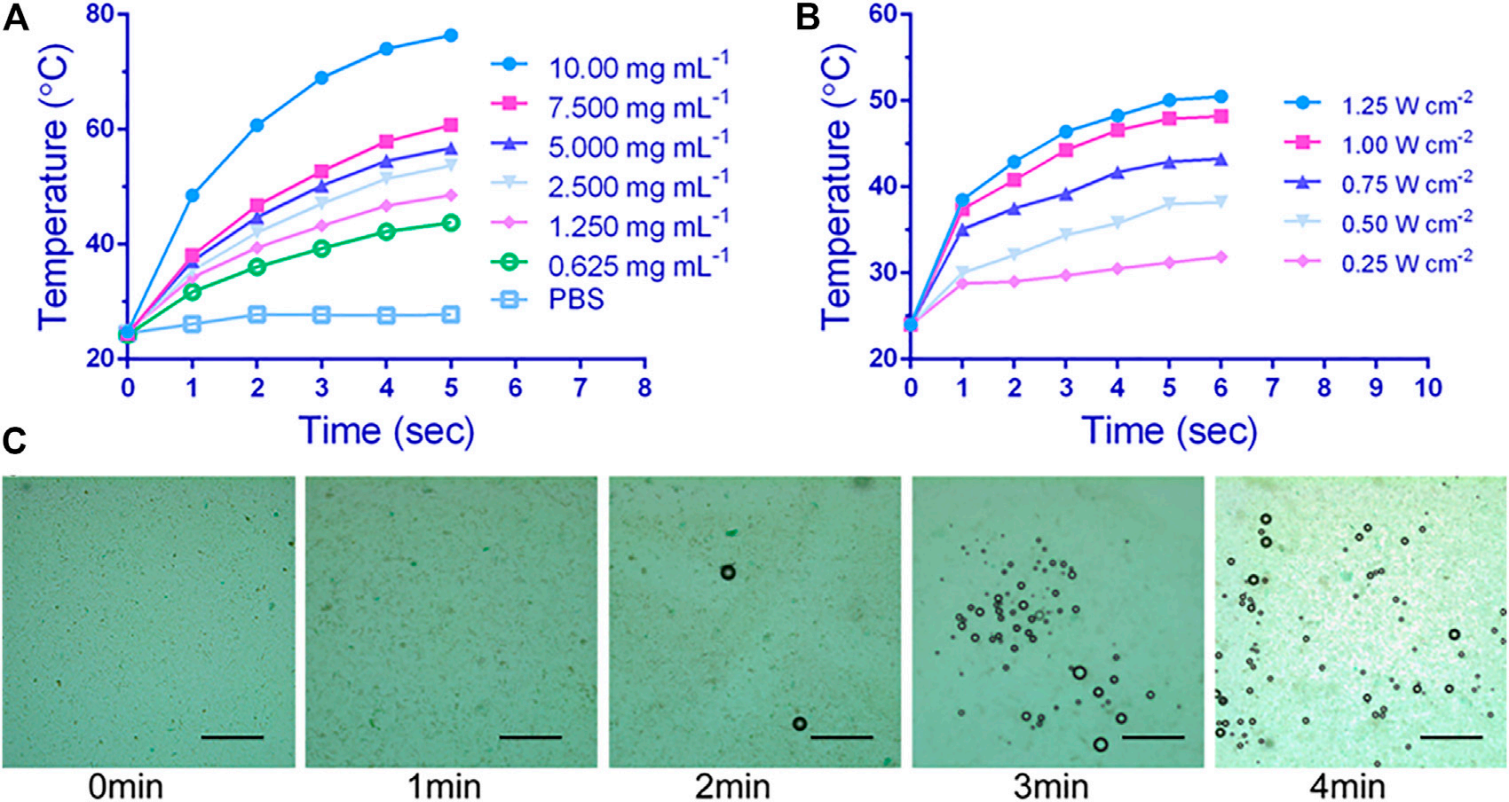
*In vitro* photothermal performance and near-infrared (NIR)-controlled phase-transition assessment. Concentration-dependent **(A)** and laser intensity-dependent **(B)** photothermal performance of P-PIP NPs, **(C)** Microscopic images of P-PIP NPs (1 mg mL^−1^) of phase-transition under 808 nm NIR laser irradiation. Scale bar: 50 μm.

The *in vitro* photothermal properties of P-PIP NPs were depended on the NPs concentration and intensity of irradiation laser. When the concentration of P-PIP NPs was 7.5 μg mL^−1^, it quickly reached above 60°C by 808 nm laser irradiation (Figure 2A). Interestingly, the increase of aqueous photothermal temperature of P-PIP NPs can be manipulated by changing the laser power (Figure 2B). As shown in Figure 2C, much more P-PIP NPs were gradually transformed into gaseous microbubbles as the temperature increased due to the prolonged laser irradiation time. These results indicated that IR780 could effectively absorb NIR light energy, causing PFP in PIP NP to become microbubbles, which can be used for subsequent ultrasound-enhanced imaging.

### 3.3 Targeting efficiency*in vitro*

**FIGURE 3:**
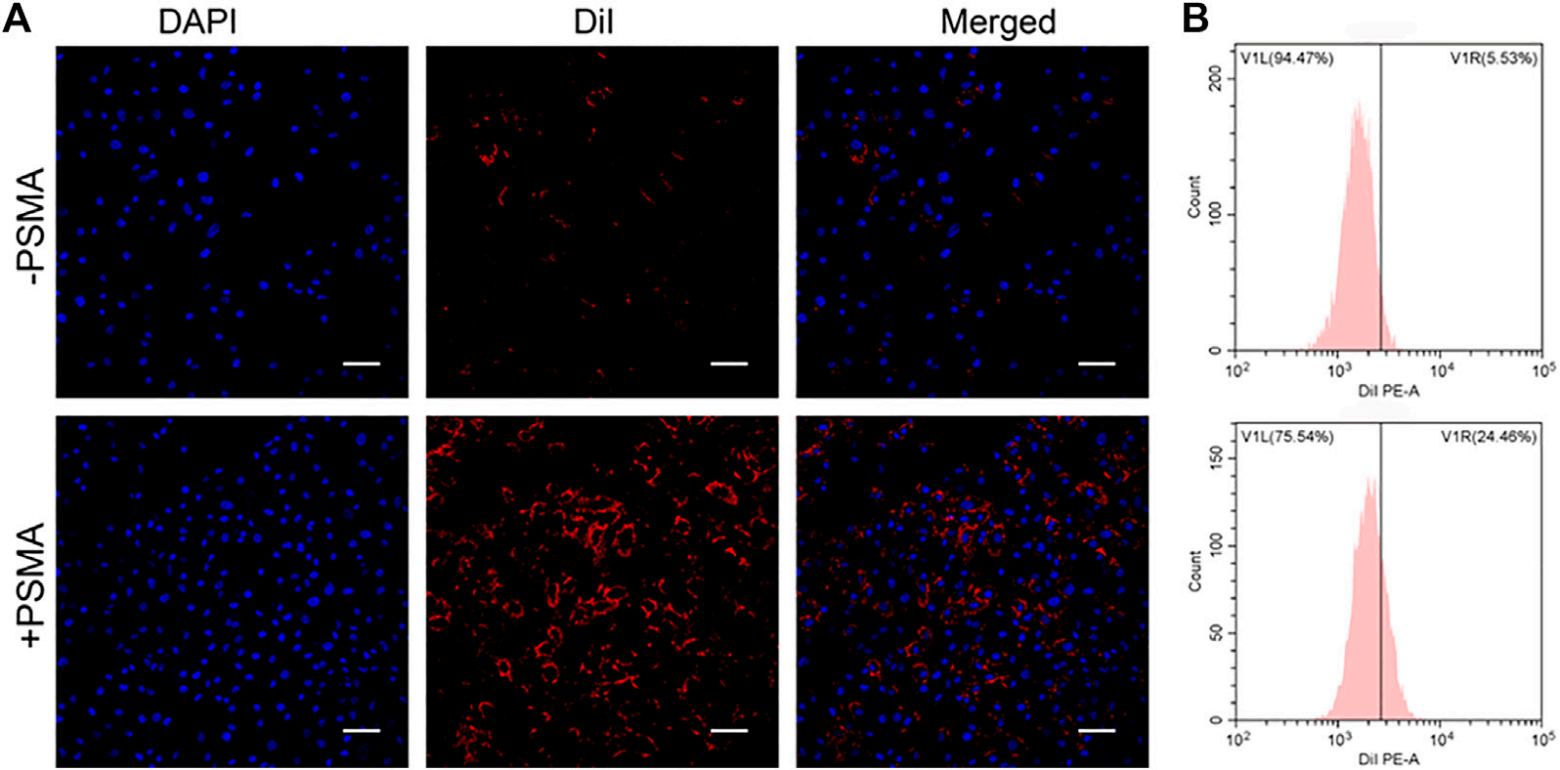
Targeting efficiency *in vitro*. **(A)** CLSM images of C4-2 cells after targeted NPs and non-targeted NPs (1 mg mL^−1^). **(B)** Flow-cytometry results of targeting efficiency. Scale bar: 50 μm.

Compared with non-targeting, PSMA-modification P-PIP NPs groups targeted C4-2 cells better, showing stronger red fluorescence in Figure 3A. In addition, as shown in Figure 3B, the results of FCM further showed that the mean fluorescence intensity of the targeted group (24.46%) was significantly higher than that of the non-targeted group (5.53%).

### 3.4 Cell viability and apoptosis assay

**FIGURE 4:**
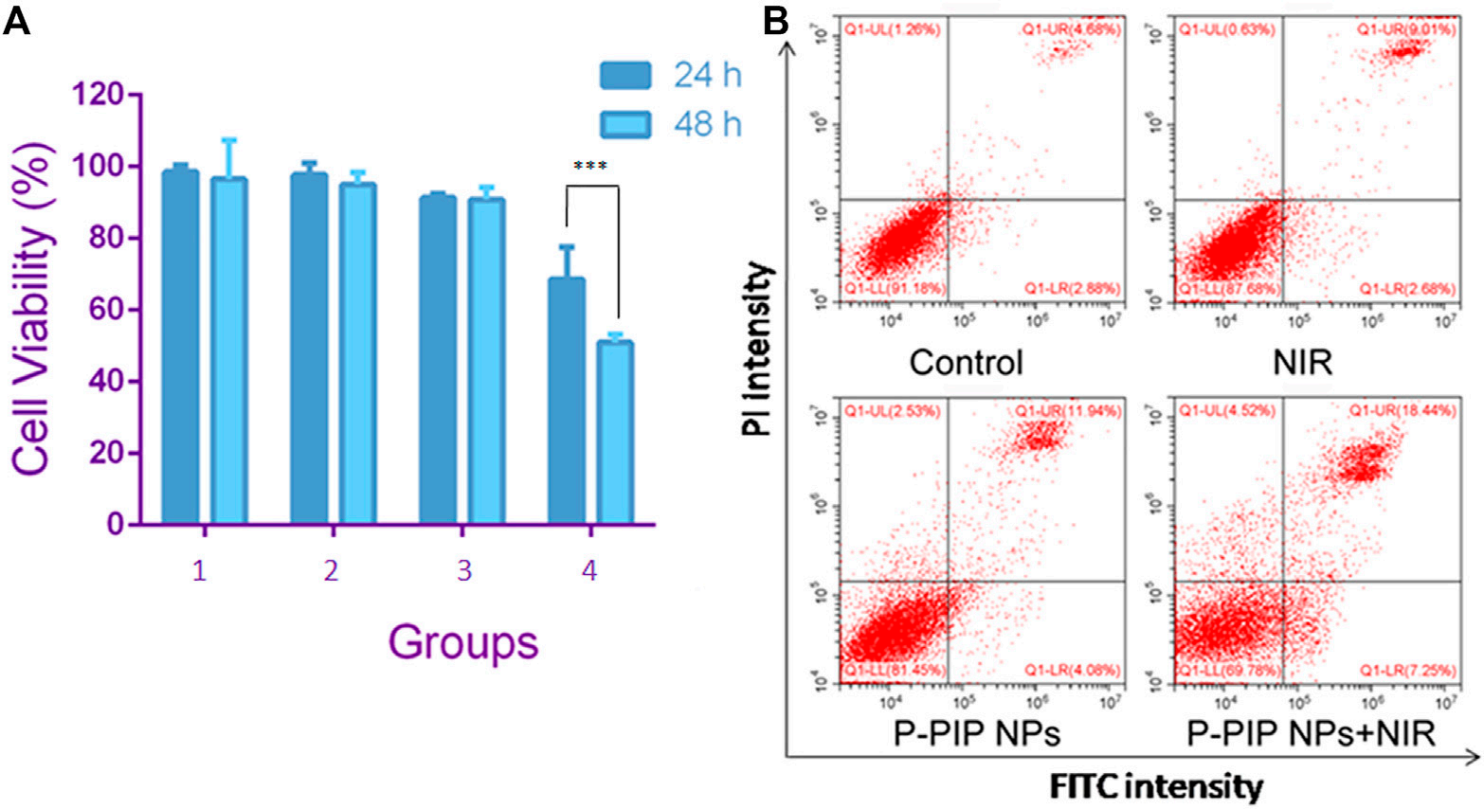
Cell viability and apoptosis assay. **(A)** Cell viability by CCK-8 assay.1: Control group; 2: NIR group; 3:P-PIP NPs group; 4: P-PIP NPs + NIR group. (***p < 0.001) **(B)** Flow-cytometry results of apoptosis.

The effect of various treatments on the cell viability of prostate cancer cell line C4-2 was assessed by CCK-8 assay. Compared with the control, NIR irradiation had little effect on cell viability. Cell viability was slightly less after P-PIP NPs treatment P-PIP NPs combined with NIR irradiated cell viability significantly decreased (Figure 4A). These results indicated that the treatment strategy of PTT combined with chemotherapy had a significant inhibitory effect on C4-2 cells and had potential application value in combined tumor therapy.

We detected apoptosis by flow cytometry on the cells after different treatments with PI and Annexin V staining. Consistent with the results of CCK-8, we found that the P-PIP NPs combined with the NIR group had the highest early and late apoptosis rates, indicating that this group had the most substantial pro-apoptotic effect on tumor cells (Figure 4B).

### 3.5 *In vitro* and *in vivo* PA/US imaging

**FIGURE 5:**
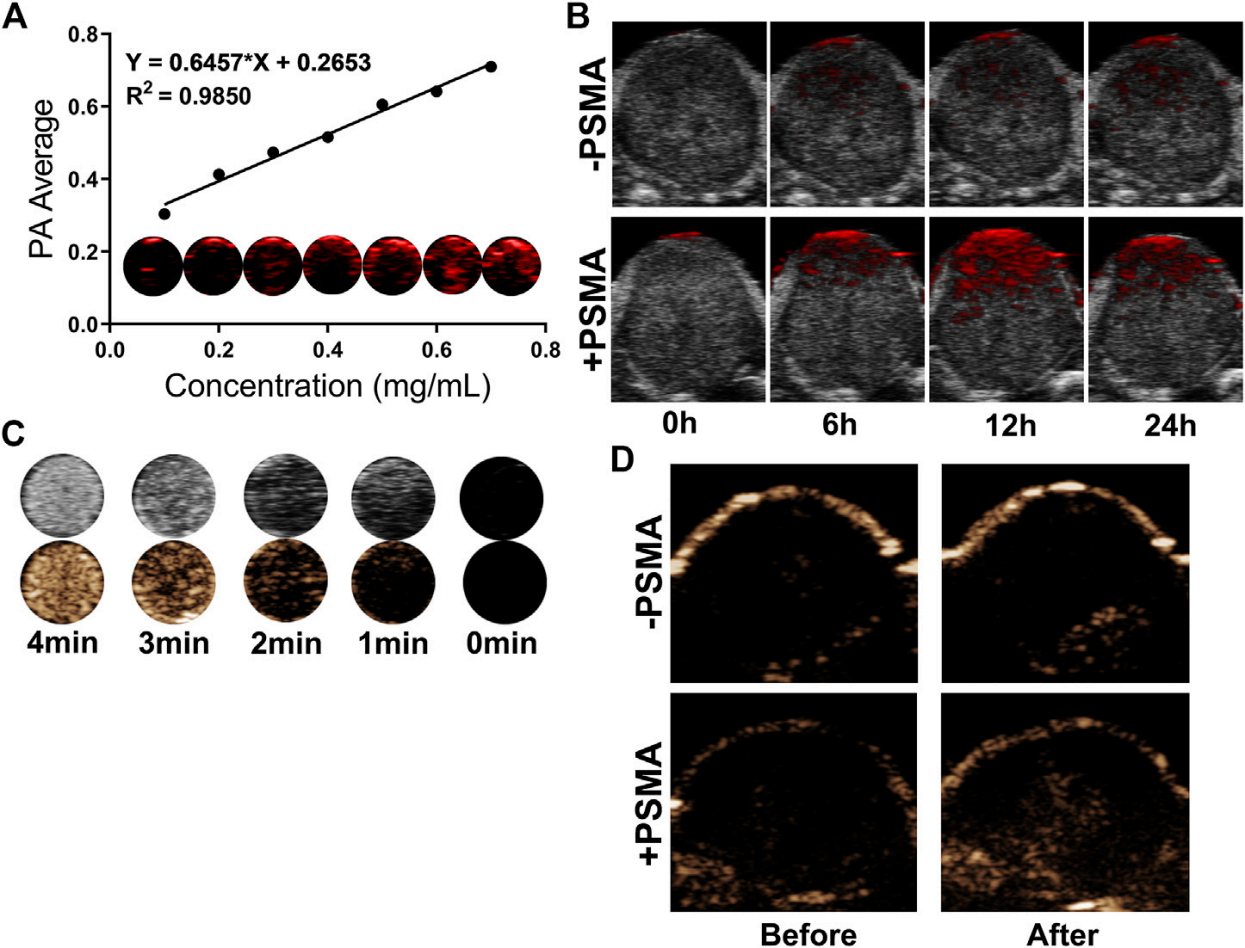
*In vitro* and *in vivo* PA/US Imaging. **(A)** *In vitro* PA images and average intensity. **(B)** PA images of tumors in C4-2 tumor-bearing mice after injection of P-PIP NPs or PIP NPs at different time points (0h, 6h, 12h and 24 h). **(C)** *In vitro* ultrasound images of different irradiation times. **(D)** contrast-enhanced ultrasound images of tumors in C4-2 tumor-bearing mice after injection of P-PIP NPs or PIP NPs.

PA/US Imaging of NPs were carried out *in vitro* and *in vivo*. First, we examined the *in vitro* imaging ability of P-PIP NPs, and found that the photoacoustic signal gradually increased and showed a linear relationship with the increase of the concentration (Figure 5A). Next, PA imaging *in vivo* was divided into targeted group and non-targeted group. It was found that tumors in the PSMA-targeted group had stronger PA signals than that of non-targeted group (Figure 5B).

*In vitro* ultrasound imaging showed that with the increase of time and temperature, more and more PFP of P-PIP NPs under near-infrared radiation underwent phase transition, which can be monitored in contrast mode (Figure 5C). Due to the targeted aggregation ability of P-PIP NPs in tumor tissues, we speculated that the phase-changeable NPs can be used as ultrasound agents to enhance ultrasound imaging to diagnose lesions. Next, ultrasound imaging *in vivo* was divided into targeted group and non-targeted group, and it was found that the tumors in the increased PSMA-targeted group had stronger US signals (Figure 5D).

### 3.6 Synergistic therapeutic ability *in vivo*

**FIGURE 6:**
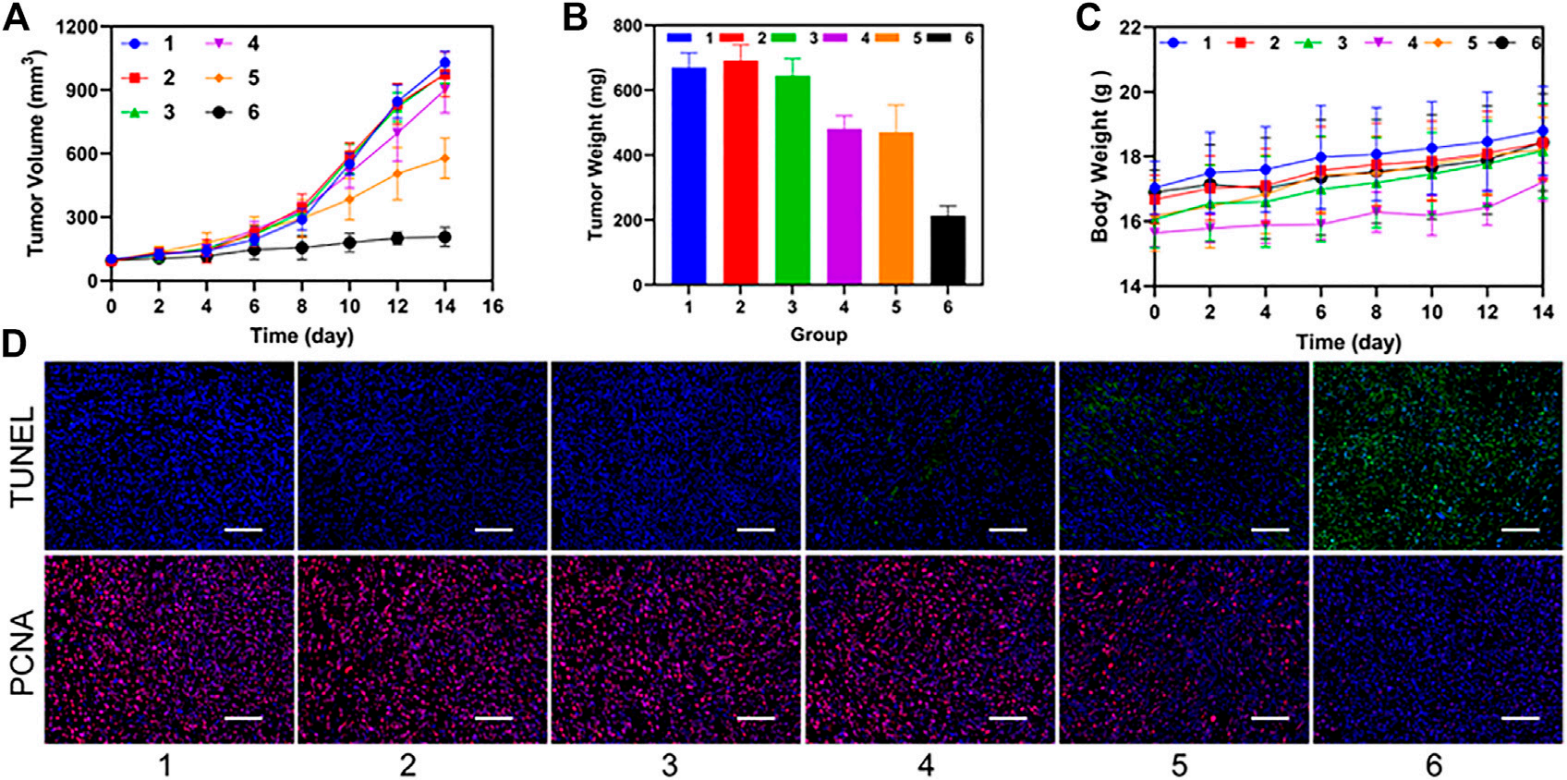
Synergistic therapeutic ability *in vivo*. **(A)** time-dependent relative tumor growth curves for the different groups. (***p < 0.001) **(B)** tumor weights in different treatment groups after treatment. (***p < 0.001) **(C)** time-dependent body weight curves of mice in the different groups. **(D)** time-dependent survival curve. f) TUNEL and PCNA staining of tumor sections after various treatments. 1: Control group; 2: NIR group; 3: PIP NPs group; 4: P-PIP NPs.; 5: PIP NPs + NIR group; 6: P-PIP NPs + NIR group. Scale bar: 50 μm.

After the treatment of C4-2 tumor bearing mice in different groups, we observed that the P-PIP NPs + NIR group had the best inhibition effect on tumor volume during the treatment period (Figure 6A). Mice were sacrificed after treatment, and tumor weight was measured, consistent with tumor volume (Figure 6B). Neither group had significant body weight changes throughout the treatment period (Figure 6C). In the pathological tissue section (Figure 6D), we found that the cells were apoptotic cells in the TUNEL staining of the tumor, and a large amount of green fluorescence was seen in the P-PIP NPs + NIR group, suggesting the strongest pro-apoptotic effect. In PCNA, cells were stained red as proliferating cells. No more prominent red fluorescence was seen in the P-PIP NPs + NIR group, indicating that proliferation was significantly inhibited.

**FIGURE 7:**
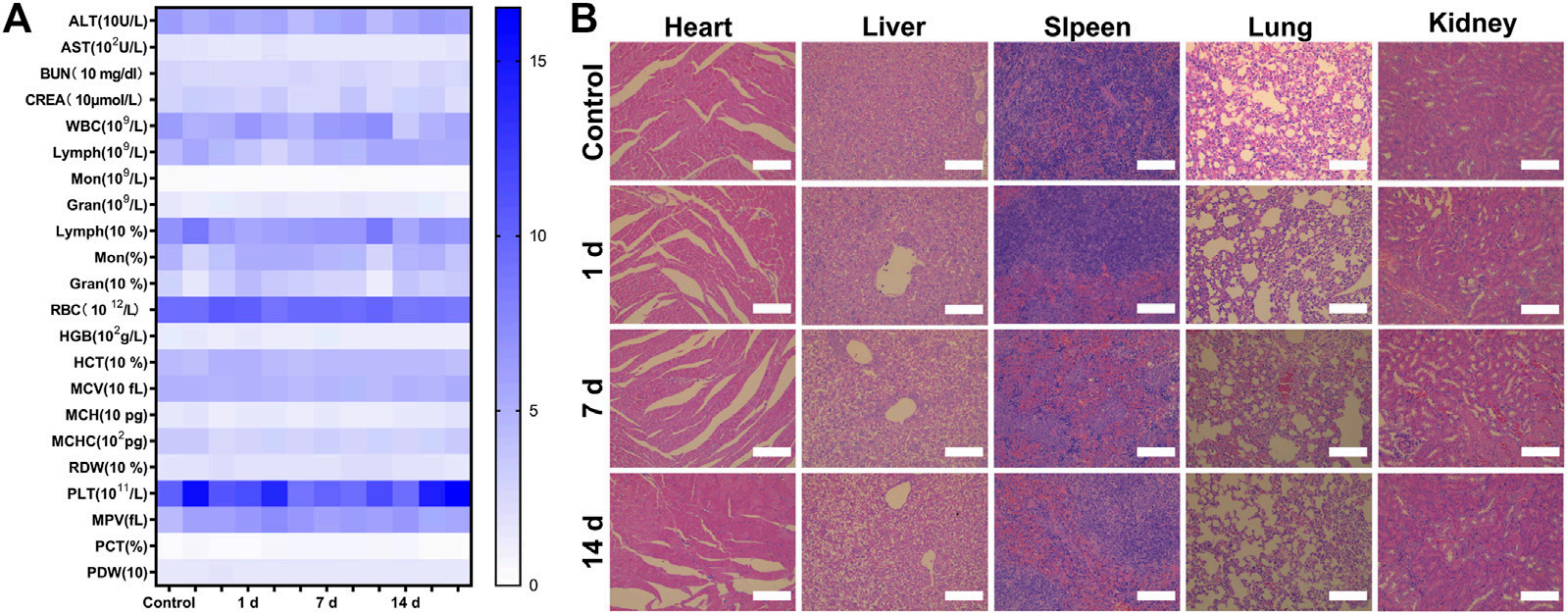
Biosafety assay of P-PIP NPs. **(A)** hematological and blood biochemical test of mice after intravenous injection of P-PIP NPs (n = 3). **(B)** H&E staining of major organs. Scale bar: 50 μm.

Finally, we evaluated the biosafety of P-PIP NPs. Compared with the control, the changes in blood routine and blood biochemical parameters were negligible at 1, 7, and 14 days after P-PIP NPs injection (Figure 7A). In addition, according to H&E staining of major organs, histopathological changes also showed that there was no toxicity of P-PIP NPs compared with the control group (Figure 7B).

## 4 Discussion

Prostate cancer (PCa) is one of the most common cancers for male (Sung et al., 2021; Liu et al., 2022). PSMA is the most attractive of prostate antigen, which is highly expressed in prostate epithelial cells (Ceci et al., 2017). Thus, PSMA has been considered as a potential target for PCa targeted diagnosis and therapy (Sun et al., 2021; Chen et al., 2022; Guo et al., 2022).

In this study, we developed photo-triggerable nanoparticles. FDA-approved PLGA was used as the shell polymer of nanocarriers. And the surface of the nanoparticles with modified by anti-PSMA antibody. We used a FCM to observe the binding between PMSA and PLGA nanoparticles and BCA protein assay to detect the efficiency of covalent conjugation between antibody and PLGA nanoparticle. This result indicated that PSMA was successfully attached to the polymer nanoparticles. In addition, the photosensitizer IR780 and phase-changeable liquid PFP were co-loaded in the PLGA nanoparticles. IR 780 enabled the nanoparticles have PA imaging and photothermal capabilities. While, PFP liquid could be triggered to have a liquid-to-gas transition by NIR irradiation resulted in the contrast-enhanced ultrasound imaging. Thus, the loading chemotherapeutic drug paclitaxel was activated released on demand. The photothermal properties of P-PIP NPs were investigated. The results showed NPs concentration and intensity of irradiation laser were the two main parameters affecting photothermal effects. NIR irradiation could heat the nanoparticles and induce PFP liquid within NPs change to gas bubbles. At the fixed nanoparticles concentrations, the number of PFP gaseous bubbles increased with the laser irradiation time.

The *in vivo* PA/US Imaging and synergistic therapeutic ability *in vivo* were tested by using tumor-bearing mice. PA imaging and US imaging were obtained. Mediated by PSMA-targeted NPs, the target tumors had stronger PA and US signals than that of non-targeted group. These results indicated that IR780 could effectively absorb NIR light energy, causing PFP in PIP NP to become microbubbles, which can be used for subsequent ultrasound-enhanced imaging as well as PA imaging. Due to the targeted aggregation ability of NPs within tumors, the phase-changeable NPs can be used as imaging probes to enhance ultrasound/PA imaging to diagnose lesions. Furthermore, the targeted NPs exhibited best inhibition effect on tumor volume during the treatment period after NIR irradiation. After H&E and TUNEL staining, the targeted groups had the strongest pro-apoptotic effect. That means the treatment strategy of PTT combined with chemotherapy had a significant inhibitory effect on PCa and had potential application value in combined tumor therapy.

## 5 Conclusion

We prepared phase-changable nanoparticles that can targeted PSMA of PCa. By loading IR780, the laser responsive nanoparticles had PA imaging and photothermal capabilities. The liquid-to-gas transition of PFP after NIR irradiation enabled contrast-enhanced ultrasound imaging and released the chemotherapeutic drug paclitaxel. This approach allowed nanosystems to have the capability of US/PA dual-modality imaging as well as precision tumor targeted therapy under the guidance of imaging.

## Data Availability

The raw data supporting the conclusions of this article will be made available by the authors, without undue reservation.
