# Supplementary material for: Laser-activated nanoparticles for ultrasound/photoacoustic imaging-guided prostate cancer treatment
Source: Front Bioeng Biotechnol. 2023 Mar 21;11:1141984. doi: 10.3389/fbioe.2023.1141984 (PMC10070956; doi:10.3389/fbioe.2023.1141984)
Supplement: Supplementary file 1 [file DataSheet1.docx]

**Supporting information**

**Laser-activated nanoparticles for ultrasound/photoacoustic imaging-guided prostate cancer treatment**

**Linkang Xiao^1,2^, Yunfang Wu ^1,3^, Junyong Dai ^1,4^,Weili Zhang^1*^, and Yang Cao^1,*^**

1 Chongqing Key Laboratory of Ultrasound Molecular Imaging, Institute of Ultrasound Imaging, Department of Urology Surgery, Second Affiliated Hospital, Chongqing Medical University, Chongqing 400010, China;

2 Chongqing General Hospital, Chongqing, 400013, China

3 Chongqing Wanzhou District Maternal and Child Health Hospital, Chongqing, 404197, China

4 Chongqing University Cancer Hospital, Chongqing, 400044, China

***Correspondence:**

Address: No. 74, Linjiang Road, Yuzhong District, Chongqing, 400010, China

Authors: Professor Weili Zhang: [300458@hospital.cqmu.edu.cn](mailto:300458@hospital.cqmu.edu.cn); Professor Yang Cao: yangcao@cqmu.edu.cn.


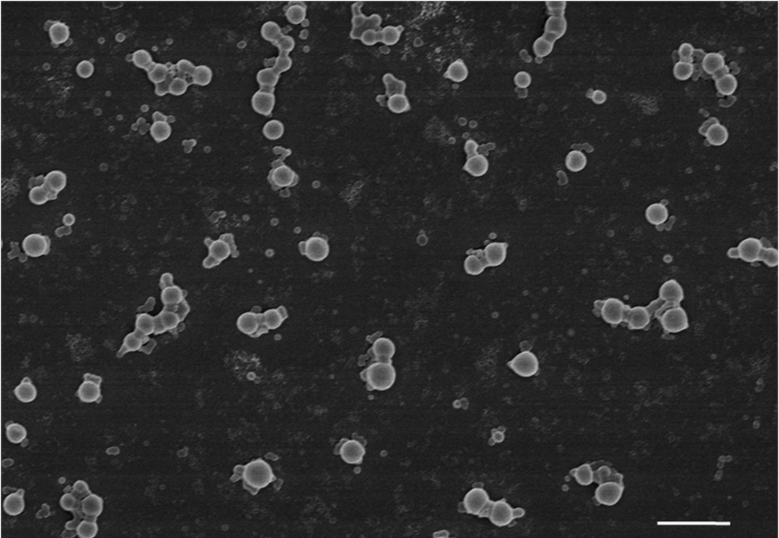


Fig.S1. SEM images of nanoparticles (Scale bar: 1 μm)


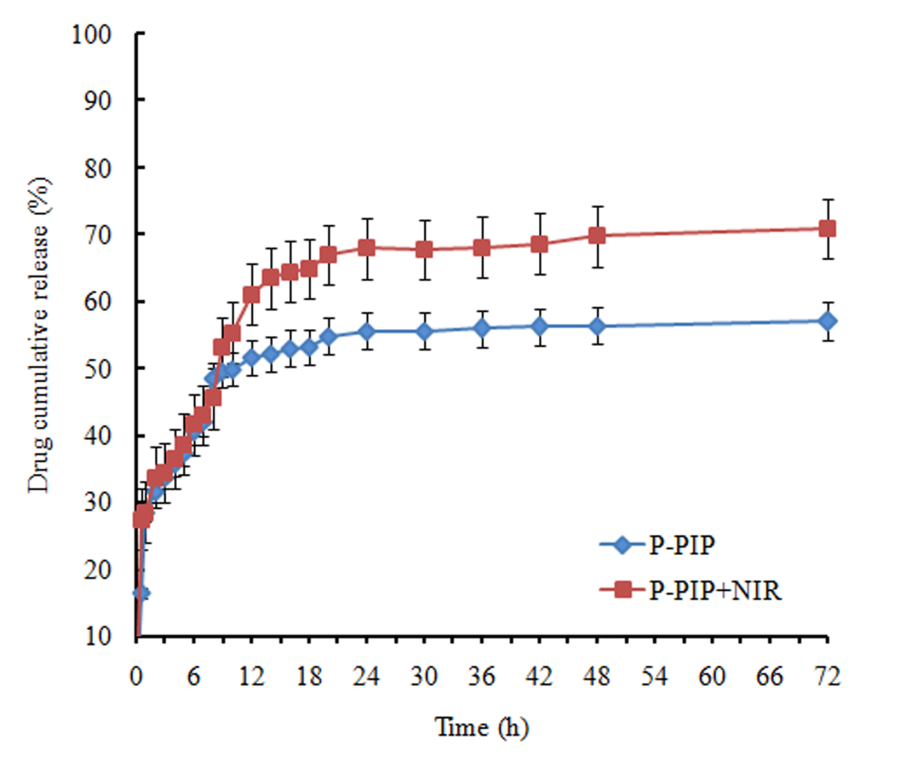


Fig.S2. In vitro drug release profiles after NIR irradiated
